# Development of Bioluminescent Virulent *Aeromonas hydrophila* for Understanding Pathogenicity

**DOI:** 10.3390/pathogens12050670

**Published:** 2023-05-02

**Authors:** Eda Ozdemir, Hossam Abdelhamed, Ozan Ozdemir, Mark Lawrence, Attila Karsi

**Affiliations:** Department of Comparative Biomedical Sciences, College of Veterinary Medicine, Mississippi State University, Starkville, MS 39762, USA

**Keywords:** *luxCDABE*, bioluminescence, vAh, ML09-119, BLI, *Ictalurus punctatus*

## Abstract

Virulent *Aeromonas hydrophila* (vAh) strains that cause motile *Aeromonas* septicemia (MAS) in farmed channel catfish (*Ictalurus punctatus*) have been an important problem for more than a decade. However, the routes of infection of vAh in catfish are not well understood. Therefore, it is critical to study the pathogenicity of vAh in catfish. To this goal, a new bioluminescence expression plasmid (pAK*gfplux*3) with the chloramphenicol acetyltransferase (*cat*) gene was constructed and mobilized into vAh strain ML09-119, yielding bioluminescent vAh (BvAh). After determining optimal chloramphenicol concentration, plasmid stability, bacteria number–bioluminescence relationship, and growth kinetics, the catfish were challenged with BvAh, and bioluminescent imaging (BLI) was conducted. Results showed that 5 to 10 µg/mL chloramphenicol was suitable for stable bioluminescence expression in vAh, with some growth reduction. In the absence of chloramphenicol, vAh could not maintain pAK*gfplux*3 stably, with the half-life being 16 h. Intraperitoneal injection, immersion, and modified immersion (adipose fin clipping) challenges of catfish with BvAh and BLI showed that MAS progressed faster in the injection group, followed by the modified immersion and immersion groups. BvAh was detected around the anterior mouth, barbels, fin bases, fin epithelia, injured skin areas, and gills after experimental challenges. BLI revealed that skin breaks and gills are potential attachment and entry portals for vAh. Once vAh breaches the skin or epithelial surfaces, it can cause a systemic infection rapidly, spreading to all internal organs. To our best knowledge, this is the first study that reports the development of a bioluminescent vAh and provides visual evidence for catfish–vAh interactions. Findings are expected to provide a better understanding of vAh pathogenicity in catfish.

## 1. Introduction

Catfish production is the largest aquaculture industry in the US, and Mississippi is the largest catfish-producing state. During the past decade, US catfish farmers produced an average of 347 million pounds of catfish per year, and Mississippi farmers accounted for 54% of total production [1]. *Aeromonas hydrophila* is a Gram-negative facultative anaerobe of the family *Aeromonadaceae* [2]. It is ubiquitous in aquatic environments and acts as an opportunistic pathogen for stressed fish, causing motile *Aeromonas* septicemia (MAS) [3,4,5].

Although *A. hydrophila* has not been a significant problem in catfish farming, the emergence of virulent *A. hydrophila* (vAh) as a primary pathogen of food-size catfish resulted in industry-wide losses in Alabama in 2009 [6]. In subsequent years, vAh spread to catfish farms in Mississippi and Arkansas [7]. It has been shown that vAh isolates were highly virulent in catfish compared to non-epidemic isolates [8]. These vAh isolates contained unique DNA sequences not present in non-epidemic isolates [9]. In addition, genome sequences revealed unique features of vAh strains [10,11]. The US vAh isolates were highly clonal and were distinct from non-epidemic *A. hydrophila* [12]. Interestingly, vAh carp isolates from China and vAh catfish isolates from the US have highly similar genomes, suggesting that US vAh may have been introduced from Asia [13]. Comparative genomic analyses revealed that vAh consists of multiple lineages with distinct features and unique conserved regions [14,15,16].

The virulence factors of *A. hydrophila* include biofilm, capsule, O-antigen, S-layer protein, secretion systems, hemolysins, degradative enzymes, enterotoxin, extracellular products, outer membrane proteins, flagella, pili, and fimbria [14,17,18,19,20,21,22,23,24,25].

Studies exploring the transmission of vAh in catfish ponds reported potential spread by fish-eating birds [26,27]. Virulent *A. hydrophila* can survive in aquatic environments for an extended period at moderate and warm temperatures [28]. Therefore, pond water temperature is essential for MAS, as most outbreaks were observed at or above 30 °C [16]. Although the natural route of infection in catfish is not well understood, skin, gill, and gastrointestinal tract are all possible routes of vAh entry [16]. Clinical signs of MAS caused by vAh include widespread hyperemia; severe hemorrhagic lesions in the skin, muscle, gills, and internal organs; exophthalmia; ascites; and extensive edema. The pathological analysis included lesions in the gill, spleen, intestine, kidney, liver, and stomach [16,29].

Initial experimental challenge methods were based on the intraperitoneal injection of vAh because immersion challenge methods did not yield consistent results. In injection challenges, vAh strains were at least 200-fold more virulent, resulting in mass mortality in less than 24 h compared to non-epidemic isolates [8,17]. Inducing stress, removing skin mucus, breaching skin integrity, and altering fish feeding and iron availability in a bacterial culture medium have yielded successful vAh infections by immersion challenge, causing mass mortalities within 48 h with lesions in internal organs [15,30,31,32,33,34].

In bacteria, the luciferase enzyme catalyzes the oxidation of reduced riboflavin phosphate and a long-chain fatty aldehyde, resulting in bioluminescent light emission. Because the *lux* operon (*luxCDABE*) encodes a luciferase enzyme and an aldehyde substrate, there is no need to add an exogenous substrate. [35]. Bioluminescence imaging (BLI) in live animal models facilitates tracking pathogens. It also allows data collection from the same host animals during infection, reducing the number of animals used and variability between time points [36,37,38]. Our group developed bioluminescent expression plasmids and successfully utilized BLI to track catfish pathogens [39,40,41,42,43,44].

The pathogenicity of vAh and routes of vAh infection in channel catfish are poorly understood. Therefore, we developed a novel bioluminescent vAh strain in this study and aimed to understand vAh pathogenicity in live channel catfish and catfish organs. We expect that visual evidence for vAh entry and dissemination in catfish will contribute to our understanding of vAh pathology.

## 2. Materials and Methods

### 2.1. Bacterial Strains, Growth Conditions, and Plasmids

Bacterial strains and plasmids used in this work are listed in Table 1. Virulent *A. hydrophila* strain ML09-119 (vAh) is a highly virulent strain and was isolated from the kidney of a diseased channel catfish during a 2009 disease outbreak in Alabama. It was cultured for 16 h at 30 °C using brain heart infusion (BHI) agar and broth (Difco, Sparks, MD). *Escherichia coli* strains were cultured for 16 h at 37 °C using Luria–Bertani (LB) agar and broth (Difco). When required, antibiotics were added to the culture medium at the following concentrations: 100 μg/mL ampicillin, 12.5 μg/mL colistin, and 6.25 μg/mL chloramphenicol.

### 2.2. Construction of pAKgfplux3

The vAh is resistant to ampicillin and sensitive to chloramphenicol. Thus, we could not use our previously developed fluorescence and bioluminescence expression vectors that carry ampicillin resistance [39,40]. Instead, we developed a new plasmid that provided chloramphenicol resistance. To achieve this, the *cat* gene was amplified from pMJH46 (Addgene plasmid #67272) using forward (aaagagctcTCGAGATTTTCAGGAGCTAAGG) and reverse (aaaactagtAGGGCACCAATAACTGCCTTA) primers carrying *sac*I and *spe*I restriction enzyme sites (underlined bases) at their 5′ ends, respectively. PCR-amplified *cat* gene was digested with *Sac*I and *Spe*I restriction enzymes and ligated into recipient pAK*gfplux*1 (Addgene plasmid # 14083) digested with the same enzymes, yielding pAK*gfplux*3. After cloning, pAK*gfplux*3 was isolated from *E. coli* DH5α and electroporated into *E. coli* donor strain SM10λ*pir*, which was used to transfer pAK*gfplux*3 into vAh by conjugal mating. After conjugation, selection on BHI agar plates with chloramphenicol and colistin resulted in bioluminescent *A. hydrophila* (BvAh). Bioluminescence was monitored using IVIS Lumina XRMS in Vivo Imaging System Series III (PerkinElmer) and/or Cytation 5 Cell Imaging Multimode Reader (BioTek).

### 2.3. Determination of Optimal Chloramphenicol Concentration

We conducted a series of experiments to determine the optimal chloramphenicol concentration to grow the BvAh strain. In the first experiment, 12 different BvAh colonies were grown in 2 mL BHI broth with 10 µg/mL chloramphenicol for 16 h at 30 °C. After washing with fresh BHI broth without chloramphenicol, these cultures were inoculated in BHI broth with the following chloramphenicol concentrations: 0, 5, 6.25, 7.5, 10, 12.5, 15, 25, 50, 75, and 100 µg/mL. Briefly, 95 µL BHI broth with chloramphenicol was transferred into wells of a 96-well plate (each concentration had 8 wells), and each well was inoculated with a 5 µL 16 h BvAh culture. Optical density (OD_600_) was measured at 0, 4, 8, 12, 16, and 20 h using Cytation 5.

In the second and third experiments, lower chloramphenicol concentration ranges (5 µg/mL to 15 µg/mL and 0.5 µg/mL to 5.5 µg/mL, respectively) were tested using the procedures described above. In addition to OD_600_ values, bioluminescence was also captured using Cytation 5 for 1 s at 30 °C. Bioluminescence values were normalized by dividing them with OD_600_ values at each time point.

### 2.4. Plasmid Stability

Six BvAh colonies were inoculated into 2 mL BHI broth with chloramphenicol (6.25 µg/mL) and grown for 16 h at 30 °C. From these cultures, 20 µL was transferred to 980 µL BHI broth with chloramphenicol (6.25 µg/mL), and after 12 h, OD_600_ and bioluminescence values were measured using Cytation 5. From this culture, 20 µL were transferred to fresh 980 µL BHI without chloramphenicol, and OD_600_ and bioluminescence were measured after 12 h using Cytation 5. This process was repeated every 12 h for 4 days.

### 2.5. BvAh Number and Bioluminescence Relationship

Eight BvAh colonies were inoculated into 2 mL of BHI broth with chloramphenicol (6.25 µg/mL) and grown for 16 h at 30 °C. From these cultures, eight separate dilution series (from 10^0^ to 10^−12^) were prepared in black 96-well plates, and bioluminescence was determined using IVIS Lumina XRMS in Vivo Imaging System Series III for 1 m at 30 °C. After obtaining bioluminescence values, bacteria from three dilution series were spread on BHI agar with chloramphenicol (6.25 µg/mL) to determine viable BvAh numbers.

### 2.6. Growth of BvAh and vAh

Six BvAh and six non-bioluminescent vAh colonies were inoculated in 2 mL of BHI broth without chloramphenicol. In addition, 6 BvAh colonies were inoculated in 2 mL of BHI broth with chloramphenicol (6.25 µg/mL). Cultures were grown in a shaker incubator (180 rpm) for 16 h at 30 °C, and OD_600_ values were determined using a spectrophotometer (Thermo Fisher Scientific). Three different 100 mL media, two without and one with chloramphenicol (6.25 µg/mL), were inoculated (1:1000 dilution) with corresponding cultures and grown as described above. OD_600_ values were measured every 4 h for 24 h.

### 2.7. Imaging of BvAh in Live Catfish

Fifteen specific-pathogen-free (SPF) channel catfish fingerlings (15.60 ± 0.99 cm, 11.40 ± 0.94 g) were obtained from the fish hatchery of the College of Veterinary Medicine at Mississippi State University and stocked into three 40 L tanks (five fish each tank) with a continuous water flow (1 L/min) and aeration. Fish were acclimated for a week before experimental challenges. Water temperature (30 °C), light–dark cycle (12 h), chlorine, and dissolved oxygen were monitored, and fish were fed twice a day. The first group of fish was injected intraperitoneally with 100 µL of BvAh (4.57 × 10^6^ CFU) [12]; the second group was immersed in water containing 4.57 × 10^10^ CFU/mL BvAh for 3 h [19]; and the third group was infected similar to the second group after clipping off the adipose fin.

BLI was conducted at 1, 3, 6, 12, 18, 24, and 36 h post-infection using an IVIS Lumina XRMS Imaging System. Briefly, live fish were anesthetized using 100 mg/L tricaine methanesulfonate (MS222) for 3–5 min and placed in the imaging chamber (set at 30 °C) along with dead fish at that time point. Bioluminescence was captured from the left and right sides of all fish using the same parameters for 15 s. Following imaging, live fish were returned to well-aerated water for recovery, and dead fish were discarded. Bioluminescence from live fish and background was quantified using Living Image Software v 4.2, and background bioluminescence was subtracted from fish values.

### 2.8. Estimation of BvAh Quantities in Catfish Organs

Thirty-six SPF channel catfish (16.25 ± 1.04 cm, 11.90 ± 0.98 g) were acclimated for one week, and three different challenge experiments, each with twelve fish, were conducted as described above. After euthanizing three fish per time point in 350 mg/L MS-222, stomach and intestine, gills, anterior kidney, posterior kidney, liver, and spleen were collected aseptically at 6, 12, 18, and 24 h, and bioluminescence emitted from the organs was captured for 15 s at 30 °C. After BLI, the posterior kidneys, a commonly used organ providing an accurate representation of the bacterial load in the fish, were homogenized in 1000 µL of PBS, and 25 µL homogenate from 10^−1^, 10^−2^, and 10^−3^ dilutions were spread on BHI agar plates with chloramphenicol. Bioluminescence of the colonies was determined using an IVIS Lumina XRMS in Vivo Imaging System (15 s at 30 °C).

### 2.9. Statistical Analysis

We used SAS 9.4 (SAS Institute Inc., Cary, NC, USA) or SPSS V19 (IBM Corp., Armonk, NY, USA) to conduct statistical analysis. OD, RLU, and CFU values were analyzed using one-way ANOVA, and a pairwise comparison of the means was made using Tukey or Games–Howell tests (*p* < 0.05). A similar analysis was conducted for photon counts after the log_10_ transformation. The correlation between photon counts and bacteria numbers was calculated by Microsoft Excel (Microsoft Corp., Redmond, WA, USA).

## 3. Results

### 3.1. Construction of pAKgfplux3

**Figure 1:**
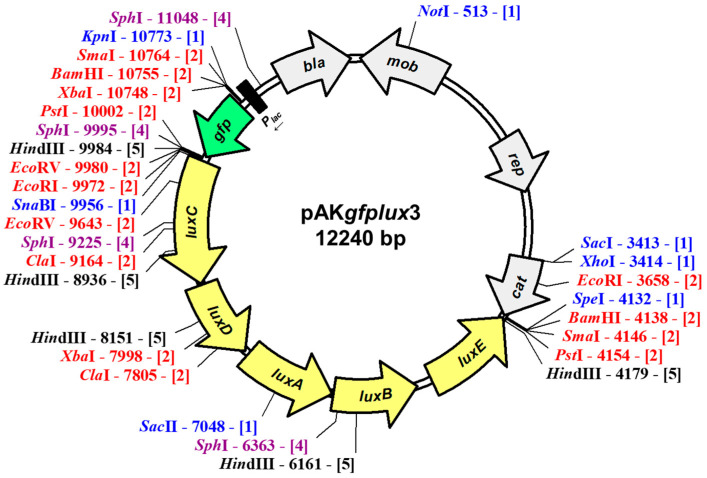
Physical map of pAK*gfplux*3 plasmid. Numbers next to restriction enzymes indicate restriction enzyme locations, while numbers in square brackets indicate the number of cuts. The map was prepared using pDRAW32 DNA analysis software (http://www.acaclone.com, accessed on 1 February 2023).

A 717 bp fragment containing a chloramphenicol resistance gene was amplified from pMJH46 and inserted into the broad host range plasmid pAK*gfplux*1, yielding a new plasmid named pAK*gfplux*3 (Figure 1). This new plasmid is 12,240 bp, and it contains green fluorescent protein (*gfp*) and bacterial luciferase (*luxCDABE*) genes expressed from the *lacZ* promoter. The plasmid pAK*gfplux*3 carries *bla* (providing ampicillin resistance) and *cat* (providing chloramphenicol resistance) genes, allowing for the expression of fluorescence and bioluminescence in ampicillin- or chloramphenicol-sensitive Gram-negative bacteria. The new plasmid has been deposited to Addgene and is available under plasmid # 199304.

### 3.2. Determination of Optimal Chloramphenicol Concentration

**Figure 2:**
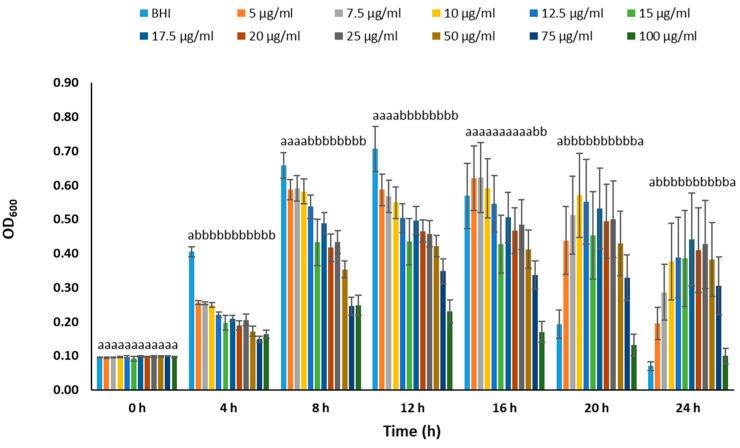
Growth of BvAh in BHI broth with 0–100 µg/mL chloramphenicol concentrations. The letters above the bars indicate statistical significance. The means of ODs were calculated from eight technical replicates.

**Figure 3:**
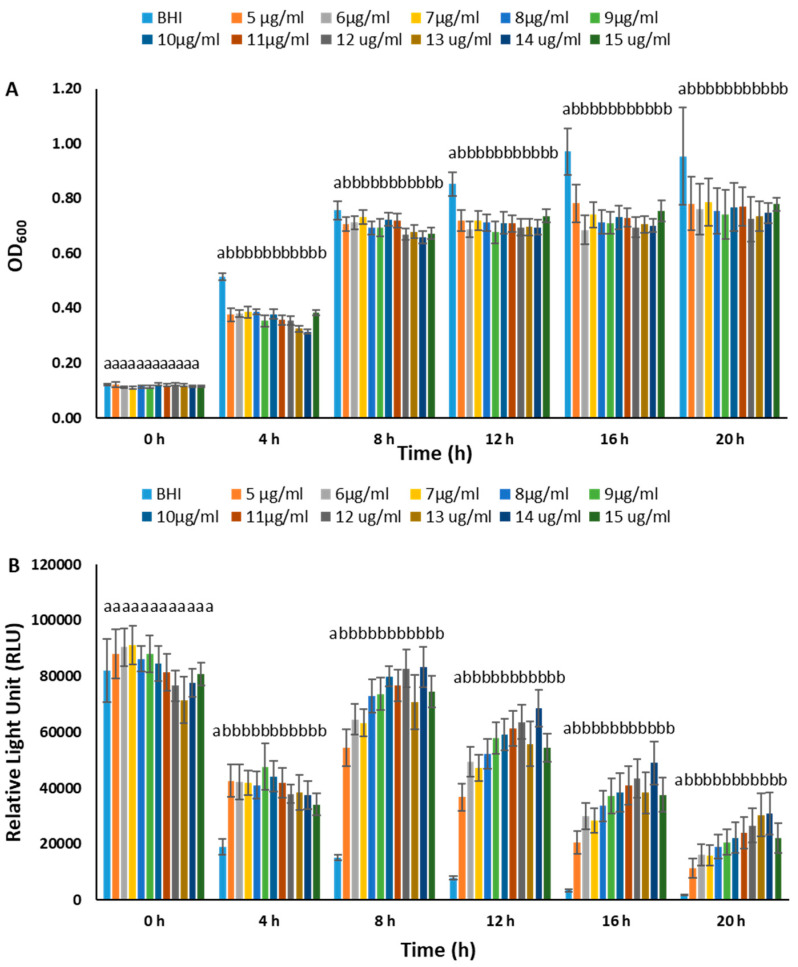
Growth (**A**) and bioluminescence (**B**) of BvAh in BHI broth with 0–15 µg/mL chloramphenicol concentrations. The letters above the bars indicate statistical significance. The means of ODs and RLUs were calculated from eight technical replicates.

**Figure 4:**
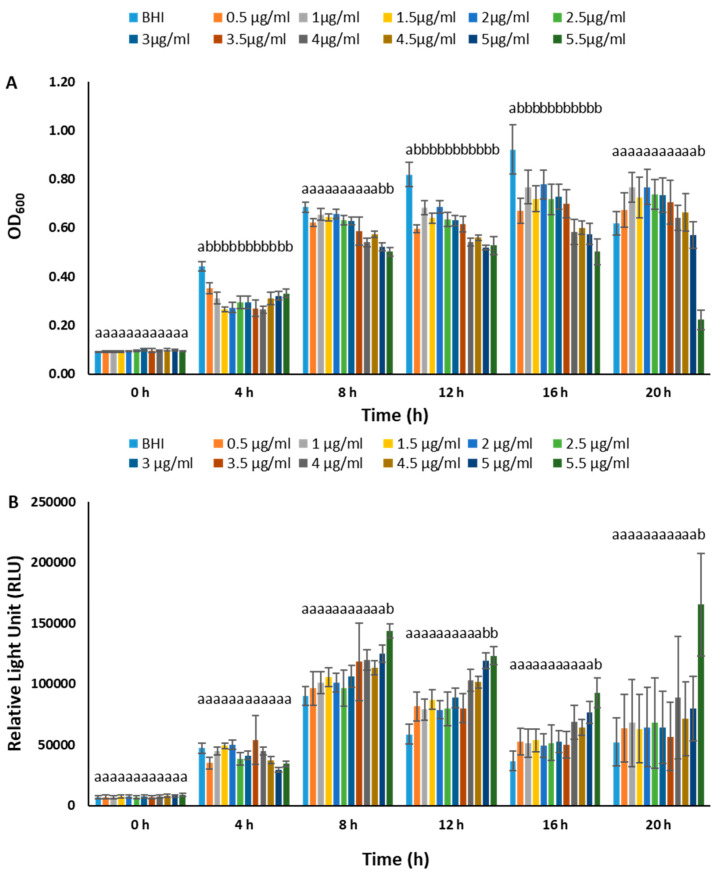
Growth (**A**) and bioluminescence (**B**) of BvAh in BHI broth with 0–5.5 µg/mL chloramphenicol concentrations. The letters above the bars indicate statistical significance. The means of ODs and RLUs were calculated from eight technical replicates.

We conducted experiments to determine the optimal chloramphenicol concentration for maintaining plasmids in BvAh. In the range of 0–100 µg/mL, the presence of chloramphenicol generally inhibited BvAh growth in a dose-dependent manner during the exponential growth phase. However, concentrations of up to 10 µg/mL yielded comparable results (Figure 2). The dose effect was similar in the 0–15 µg/mL range, although the absence of chloramphenicol led to the highest growth and lowest bioluminescence. The presence of up to 10 µg/mL of chloramphenicol increased bioluminescence, although the overall bioluminescence decreased over time (Figure 3). We observed similar trends in the 0–5.5 µg/mL range, with 5.5 µg/mL yielding the highest bioluminescence (Figure 4). Our results suggest that chloramphenicol concentrations of 5–10 µg/mL, preferably closer to the lower end of the range, are suitable for maintaining bioluminescence expression with some growth reduction. Therefore, we used a 6.25 µg/mL concentration of chloramphenicol in the subsequent experiments.

### 3.3. Stability of pAKgfplux3 in BvAh Strain

**Figure 5:**
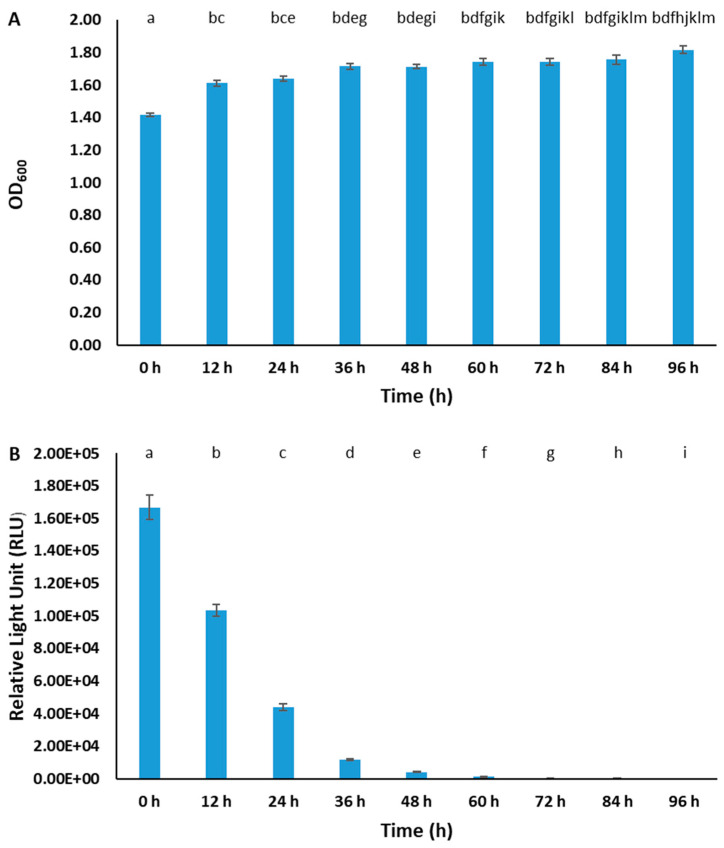
Optical density (**A**) and bioluminescence (**B**) of BvAh without chloramphenicol. Only 0 h included chloramphenicol for capturing the highest bioluminescence at the measurements’ beginning. The letters above the bars indicate statistical significance. The means of ODs and RLUs were calculated from six biological replicates.

The stability of pAK*gfplux3* was determined by subculturing BvAh every 12 h without chloramphenicol selection for 4 days. At 0 h, slightly lower growth was observed because the culture medium included chloramphenicol for achieving maximum bioluminescence. At other time points, bacterial growth was similar, but emitted bioluminescence decreased dramatically over time by 38.32% at 12 h, by 73.77% at 24 h, and by 99.05% at 60 h (Figure 5). Based on this, the half-life of the plasmid in BvAh was approximately 16 h under non-selective conditions.

### 3.4. BvAh Number and Bioluminescence Relationship

**Figure 6:**
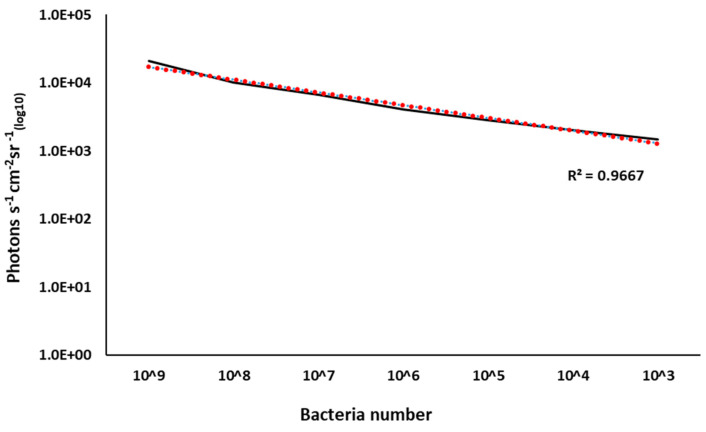
Correlation between bacteria numbers and bioluminescence in BvAh. The means of photons were calculated from eight biological replicates, and the means of bacteria numbers were calculated from three biological replicates.

The relationship between CFU and bioluminescence (photon emissions) was linear (R^2^ = 0.97) between 10^9^ to 10^3^ CFUs, and the minimum detectable number of BvAh was less than 2700 CFU/mL (Figure 6).

### 3.5. Growth of BvAh and vAh

**Figure 7:**
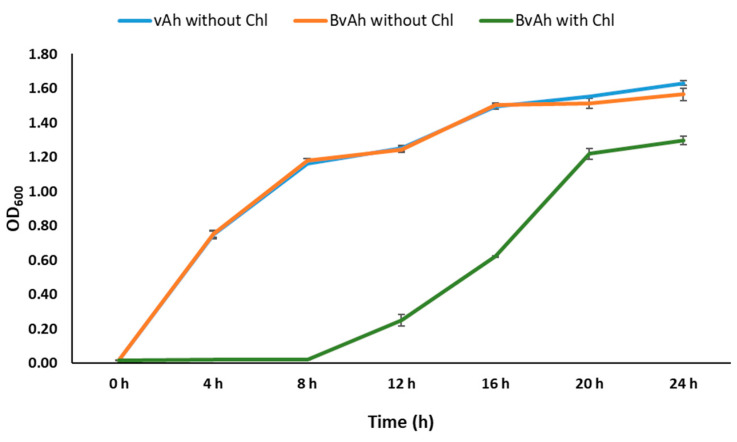
The effect of pAK*gfplux*3 and chloramphenicol on BvAh growth kinetics. The means of ODs were calculated from six biological replicates.

To investigate the effect of pAK*gfplux*3 and chloramphenicol on bacterial growth, we compared the growth of vAh without chloramphenicol and BvAh with and without chloramphenicol under identical conditions. Our results showed that the presence of pAK*gfplux*3 did not affect the growth of BvAh, but the presence of chloramphenicol caused a growth delay of 8 h in BvAh (Figure 7) (*p* < 0.05).

### 3.6. Imaging of BvAh in Live Catfish

**Figure 8:**
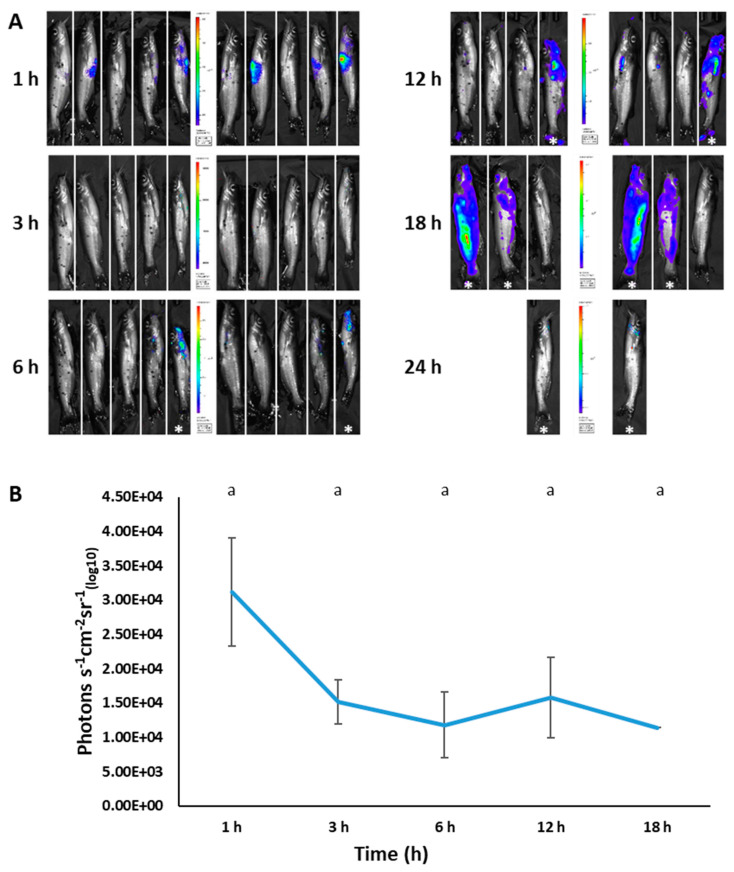
BLI of injection-challenged catfish (4.57 × 10^6^ CFU/100 µL) at different time points (**A**) and corresponding photon emissions (**B**). The dead fish (marked with an asterisk) were imaged, but their bioluminescence was not included in photon emissions. The letters above the bars indicate statistical significance. The numbers of biological replicates (live fish) used to calculate photon emissions at each time point were as follows: 1 h (5), 3 h (5), 6 h (4), 12 h (3), and 18 h (1).

We successfully used bioluminescent imaging to detect BvAh in injected and immersed fish. For each imaging time, both live and dead fish were imaged (the latter marked with an asterisk), but only photon emissions from live fish were included in the analysis. In vivo bioluminescence was observed in the abdominal area of most fish as early as 1 h post-injection. In addition, live and dead fish showed bioluminescence in various body areas, including in the mouth, barbels, pectoral fins, anterior kidney, caudal fin, lateral line, anal fin, and in the entire body (Figure 8A). Although bioluminescence decreased at 3 and 6 h post-injection, it increased again at 12 h before decreasing at the final imaging time of 18 h (Figure 8B).

**Figure 9:**
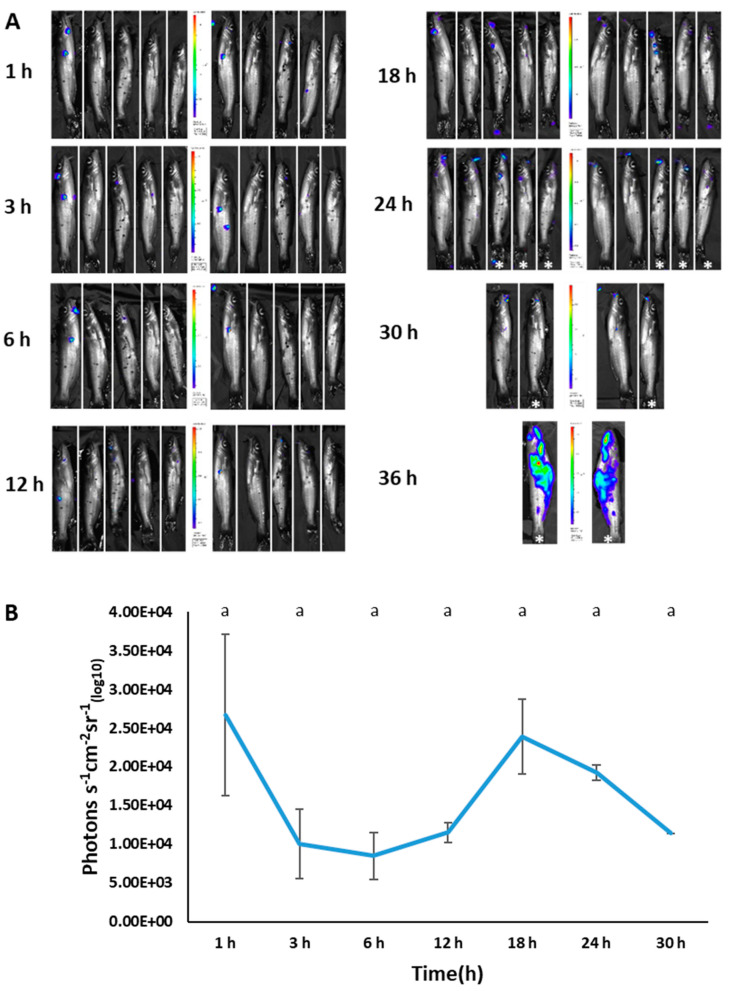
BLI of immersion-challenged catfish (4.57 × 10^10^ CFU/mL for 3 h) at different time points (**A**) and corresponding photon emissions (**B**). The dead fish (marked with an asterisk) were imaged, but their bioluminescence was not included in photon emissions. The letters above the bars indicate statistical significance. The numbers of biological replicates (live fish) used to calculate photon emissions at each time point were as follows: 1 h (5), 3 h (5), 6 h (5), 12 h (5), 18 h (5), 24 h (2), and 30 h (1).

In the immersion group, bioluminescence was visible as small patches at different body locations, including barbels, operculum, and caudal fin (Figure 9A). Total photon emissions declined up until 6 h, then increased up until 18 h; this was followed by a decline by the 30 h mark (Figure 9B).

**Figure 10:**
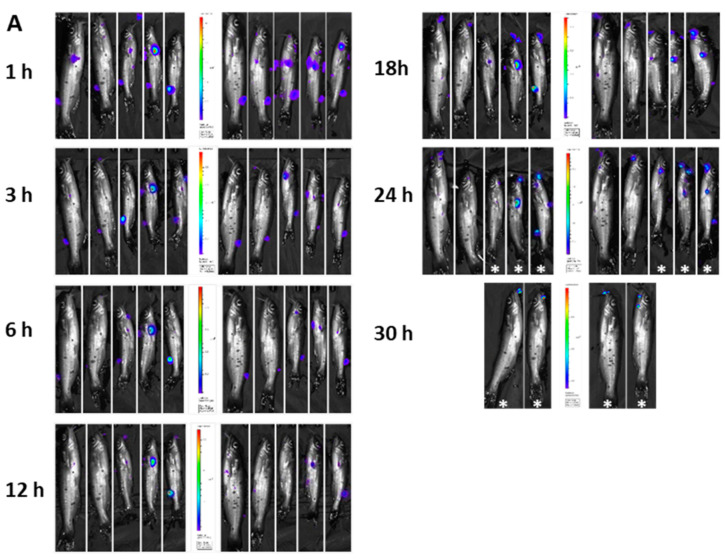

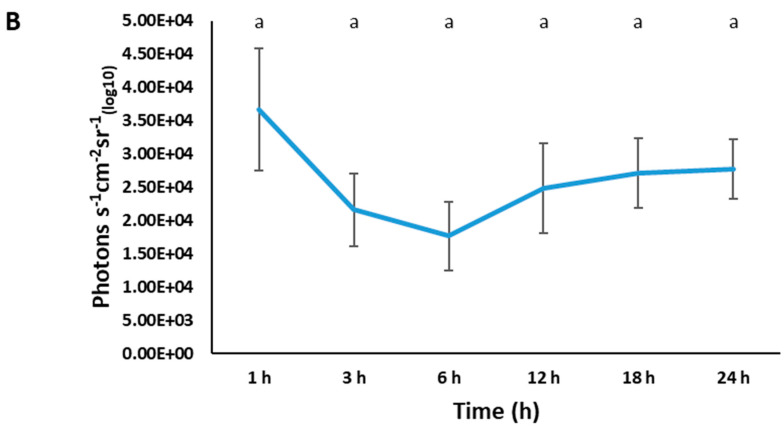
BLI of modified immersion-challenged (adipose fin-clipped) catfish (4.57 × 10^10^ CFU/mL for 1 h) (**A**) and corresponding photon emissions (**B**). The dead fish (marked with an asterisk) were imaged, but their bioluminescence was not included in photon emissions. The letters above the bars indicate statistical significance. The numbers of biological replicates (live fish) used to calculate photon emissions at each time point were as follows: 1 h (5), 3 h (5), 6 h (5), 12 h (5), 18 h (5), and 24 h (2).

In the modified immersion (adipose fin-clipped) group, all fish exhibited bacterial attachment at the clipped site at early time points (Figure 10A). Similar to the previous two experiments, total photon emission declined up until 6 h, then increased steadily until the 24 h mark (Figure 10B).

### 3.7. Estimation of BvAh Quantities in Catfish Organs

**Figure 11:**
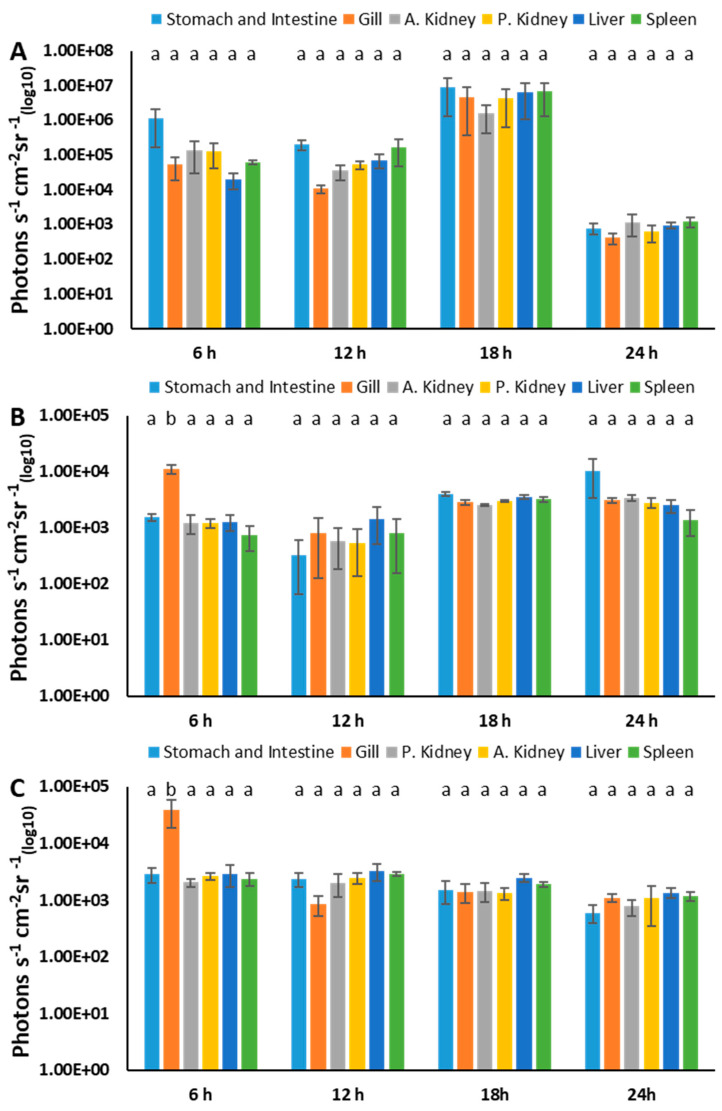
BLI of internal organs of catfish infected by injection (**A**), immersion (**B**), and modified immersion (adipose fin-clipped) methods (**C**). The letters above the bars indicate statistical significance. The means of photons were calculated from three biological replicates.

In the injection group, the bioluminescence from the stomach and intestine, anterior kidney, posterior kidney, liver, spleen, and gills did not change much at 6 and 12 h, while it increased at 18 h and decreased at 24 h (Figure 11A). In the immersion group, bioluminescence was highest in the gills at 6 h (*p* < 0.05) and increased slightly in all organs over time. Although the liver emitted higher bioluminescence at 24 h, this was not statistically significant (*p* > 0.05) (Figure 11B). In the modified immersion group, the highest bioluminescence was detected in the gills at 6 h (*p* < 0.05), while the bioluminescence of all organs decreased slightly over time (Figure 11C). Overall, the bioluminescence of the organs in the injection group was higher (>1.00 × 10^4^ photons s^−1^ cm^−1^ sr^−1^ (_log10_)) than that of both immersion groups at 6, 12, and 18 h, while it was comparable (<1.00 × 10^4^ photons s^−1^ cm^−1^ sr^−1^ (_log10_)) in all groups at 24 h.

**Figure 12:**
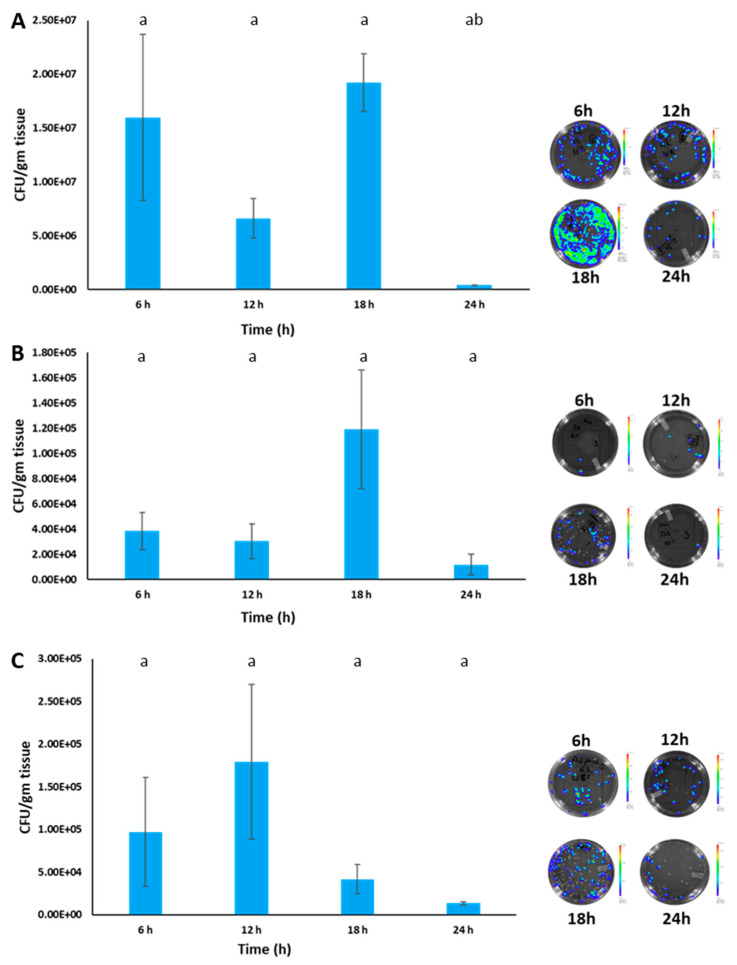
Bacterial loads in the posterior kidney of catfish from the injection (**A**), immersion (**B**), and modified immersion (adipose fin clipping) (**C**) groups. A representative agar plate with BvAh colonies at each time point is shown on the right of each graph. The letters above the bars indicate statistical significance. The means of bacteria numbers were calculated from three biological replicates.

Bacteria numbers in the posterior kidney showed a similar trend to observed bioluminescence, except that colony numbers decreased after 12 h in the modified immersion group. Bacteria numbers in the injection group were higher than in both immersion groups, and colonies recovered from the posterior kidney were bioluminescent (Figure 12A–C).

## 4. Discussion

This work aimed to develop a novel bioluminescent vAh (BvAh) and understand vAh pathogenicity in live catfish and their internal organs. Although our group developed several broad host range bioluminescence and fluorescence expression plasmids to label bacterial pathogens [39,40], we could not use them because they carry the *bla* gene, and the vAh strain ML09-119 is ampicillin resistant. The novel broad host range plasmid pAK*gfplux*3 expresses fluorescence and bioluminescence and provides ampicillin and chloramphenicol resistance, which can be a valuable tool for labeling and tracking other Gram-negative bacteria.

Virulent *A. hydrophila* stably maintained pAK*gfplux*3 under the selective pressure of chloramphenicol. However, adding chloramphenicol during BvAh growth resulted in dose-dependent slower growth. It is possible that the *cat* gene in pAK*gfplux*3 may not be expressed efficiently in vAh, causing slower growth. In addition, segregational instability may lead to plasmid-free bacteria, which may be killed by chloramphenicol in a selective environment, causing an overall slow growth of plasmid-containing bacteria.

Without the selective pressure of chloramphenicol, vAh could not maintain this broad host range plasmid stably (16 h half-life). In contrast, a plasmid with the same backbone had a half-life of 18 days in *E. ictaluri* [40] and 7 days in *Salmonella* [47]. Similar to our findings, low stability was observed in *A. salmonicida* [48]. Plasmid stability may be affected by the origin of replication (*ori*) incompatibility with native plasmids in the host strain. However, *A. hydrophila* strain ML09-119, used in this study, has no circular or chromosome-integrated plasmids. Segregational instability can also lead to a proportion of daughter bacteria losing the plasmid during binary fission, and daughter bacteria without plasmid may have a slight growth advantage in a non-selective environment due to a lower metabolic load for maintaining the plasmid. Over time, this leads to more plasmid-free bacteria [49]. Currently, the underlying mechanisms that lead to pAK*gfplux*3 instability in vAh are unknown.

In the selective environment, bacteria numbers and bioluminescence had a linear relationship (R^2^ = 0.97) between 10^9^ and 10^3^ CFUs, and the minimum detectable number of BvAh was less than 2,700 CFU/mL. Previously, we reported a similar linear relationship in bioluminescent *E. ictaluri* (R^2^ = 0.97) and bioluminescent *Salmonella* (R^2^ = 0.99), which carry the same plasmid backbone, and the minimum detectable bacteria numbers were comparable (<2500 CFU/mL and <1500 CFU/mL, respectively) [40,47].

Previous studies have evaluated the virulence of vAh using intraperitoneal injection, modified immersion (adipose fin clipping), and immersion [15,30,31,32,33,34]. We employed these three challenge methods to assess the usefulness of bioluminescent imaging (BLI) in evaluating vAh virulence. As seen in previous studies, all the fish in our experiment died within 48 h, with the injection group showing the fastest progression of mortality, followed by the modified immersion and immersion groups. After an initial lag phase, vAh appears to cause a systemic infection that leads to rapid fish mortality. Similar lag phases have been reported in other bioluminescent fish pathogens [40,43,48]. This initial lag in the bioluminescent signal may be due to bacterial adaptation to the adverse host environment as the fish’s immune system actively tries to eliminate the pathogen. During this period, bioluminescence production may be reduced as the bacteria enter survival mode or are killed by the fish’s innate immunity. After this lag period, the bacteria may adapt and replicate within the fish body, leading to mortality and a subsequent increase in bioluminescence. However, we observed a reduction in bioluminescence after 12 h (injection) and 18 h (immersion). This may be due to the high plasmid instability observed in vAh under non-selective conditions (with a half-life of 16 h). It is important to note that photon emissions were calculated only from live fish, and the removal of dead fish and high fish-to-fish variation in disease occurrence may also contribute to bioluminescence reduction.

BvAh was detected around the anterior of the mouth, barbels, fin bases, fin epithelia, injured skin areas, and gills after experimental challenges. These areas are common sites where the loss of epithelial integrity or skin injuries occur, providing potential attachment and entry portals for vAh. The importance of mucus on fish skin is critical for preventing pathogen entry, and one study reported that the removal of skin mucus aided an *A. hydrophila* infection in catfish [30]. Lesions around the mouth, fin bases, and gills are common in catfish infected with vAh [15], supporting our findings.

The adipose fin is not considered an essential organ [50] but probably functions as a precaudal flow sensor [51]. It was shown that the clipping of the adipose fin increased catfish susceptibility to vAh [31]. Our work showed that BvAh was attached at the adipose fin cut site, supporting the fact that the integrity of the fish’s skin is critical for the fish’s health, and skin injuries predispose fish to vAh infections.

A large number of infected fish (approximately 90%) died within 48 h after the immersion challenge, similar to previous studies [32,33]. Although the natural route of vAh infection in catfish is not well understood, skin, gill, and gastrointestinal tract are all possible routes of vAh entry [16]. A previous immersion challenge using vAh indicated that lesions appeared in the stomach and spleen after 1 h post-challenge and spread to other organs, including the intestine and gills, after 24–48 h [33]. In the current study, we observed bioluminescence from the stomach and intestine, suggesting a possible gastrointestinal entry route of vAh. In addition, both immersion challenges indicated that the bioluminescence of the gills was significantly higher than that of other organs at the early stages, providing evidence that gills are potential entry points of vAh. In general, injection caused a high vAh load in the fish organs up until 18 h compared to immersion, which correlated with the bioluminescent signal obtained from the fish body as well as from the posterior kidney. As discussed above, lower bioluminescence may be due to plasmid instability, bacterial killing by the fish immune system, and fish-to-fish variation of disease occurrence.

This new broad host range plasmid system yielded a novel bioluminescent vAh strain for the first time, and BLI studies suggest that skin integrity and epithelial and mucosal barriers are essential to avoid vAh infections. Thus, non-optimal aquaculture practices that cause skin injuries may make catfish more prone to vAh infections. In addition, a high number of skin injuries expected in older fish could likely increase their susceptibility to vAh infections.

## Figures and Tables

**Table 1:** Bacterial strains and plasmids used in this study.

Strains and Plasmids	Relevant Characteristics	References
*Aeromonas hydrophila*		
ML09-119	Wild type; Amp^r^, Col^r^; Chl^s^	[12]
*Escherichia coli*		
DH5α	F^−^; Φ80*lacZ*Δ*M15*; Δ(*lacZYA*-*argF*) *U169*; *recA1*; *endA1*; *hsdR17*(rk^−^, mk^+^); *phoA*; *supE44*; *thi-1*; *gyrA96*; *relA1*; λ^−^	ThermoFisher
SM10λ*pir*	*thiL*; *thrL*; *leuB6*; *tonA21*; *lacY1*; *supE44*; *recA*::RP4-2-Tc:*Mu*λ*pir*R6K; Km^r^	[45]
Plasmids		
pAK*gfplux*1	pBBR1MCS4; *gfpmut3a*; *luxCDABE*	[39]
pMJH46	pKD46; *oriT*; *traJ*; *traK*; *cat*	[46]
pAK*gfplux*3	pAK*gfplux*1; *cat*	This Study

Amp: ampicillin, Col: colistin, Chl: chloramphenicol, Km: kanamycin, r: resistant, s: sensitive, *gfp*: green fluorescent protein, *lux*: bacterial *luxCDABE* operon.

## Data Availability

The data supporting this study’s findings are available from the corresponding author upon reasonable request.
